# Review of Kyotorphin Research: A Mysterious Opioid Analgesic Dipeptide and Its Molecular, Physiological, and Pharmacological Characteristics

**DOI:** 10.3389/fmedt.2021.662697

**Published:** 2021-04-01

**Authors:** Hiroshi Ueda

**Affiliations:** ^1^Graduate School of Pharmaceutical Sciences, Kyoto University, Kyoto, Japan; ^2^Institute of Biomedical Sciences, Nagasaki University, Nagasaki, Japan; ^3^Research Institute for Production Development, Kyoto, Japan

**Keywords:** analgesia, pain, opioid “O” type, G protein, tyrosyl-tRNA synthetase, Alzheimer's disease

## Abstract

Tyrosine-arginine (kyotorphin), an opioid analgesic dipeptide, was discovered more than 40 years ago in 1979. The evidence accumulated during this period has established the physiological significance of kyotorphin as a neuromodulating peptide, and pharmacological applications. Some of the following important findings have been discussed in this review: (1) kyotorphin is unevenly distributed in the brain; it is found in high concentrations in the pain pathway, which involves the regions associated with morphine analgesia; (2) kyotorphin is subcellularly localized in the synaptosome fraction or nerve-ending particles; (3) a specific synthetase generates kyotorphin from tyrosine and arginine; (4) kyotorphin may be also processed from calpastatin by a novel calcium-activated neutral protease or calpain; (5) kyotorphin preloaded into the synaptosome is released by high K^+^ depolarization in a Ca^2+^-dependent manner; (6) kyotorphin has a specific G protein coupled receptor, which mediates the activation of phospholipase C (PLC) and inhibition of adenylyl cyclase through G_i_; (7) leucine-arginine works as a specific kyotorphin receptor antagonist; 8) membrane-bound aminopeptidase or excretion through a peptide transporter, PEPT2, may contribute to the inactivation of kyotorphin; and (9) kyotorphin causes increased Met-enkephalin release from brain and spinal slices. It is also known that the opening of plasma membrane Ca^2+^ channels through a conformational coupling of the InsP_3_ receptor with the transient receptor potential C1, which is downstream of the kyotorphin receptor-mediated activation of G_i_ and PLC, could be a potential underlying mechanism of Met-enkephalin release. Considering these findings, translational research is an exciting domain that can be explored in the future. As kyotorphin is a small molecule, we could design function-added kyotorphin derivatives. These studies would include not only the brain-permeable kyotorphin derivatives but also hybrid kyotorphin derivatives conjugated with small compounds that have additional pharmacological actions. Further, since there are reports of kyotorphin being involved in either the etiology or treatment of Alzheimer's disease, epilepsy, inflammation, and chronic pain, studies on the beneficial effects of kyotorphin derivatives should also be expected in the future.

## Introduction

Morphine is an active and major ingredient in opium, the dried latex extract from unripe seedpods of *Papaver somniferum*. Archeological remains and artifacts from ancient Egypt and Greece indicate that opium was used as a hypnotic or pain killer as well as for recreational purposes (1). Around 1800, Friedrich Wilhelm Adam Serturner, a German pharmacist, successfully isolated and crystallized morphine from opium (2), an achievement that could be considered the beginning of modern pharmaceutical science. During the American Revolution, opium was widely used to alleviate pain in wounded soldiers, following which morphine was widely used as an analgesic. By the late 1800s, morphine was being overused, resulting in morphine addiction. However, with the development of sustained release morphine pellets and a better understanding of the appropriate use of opioids, as recommended by the World Health Organization in 1986, opioids have become essential for pain management in cancer patients. Nevertheless, death due to opiate overuse is a major issue in several Western countries.

In the late 1960s, extensive studies were conducted to develop opiates or morphine derivatives that did not cause addiction, tolerance, and dependence. In the early 1970s, morphine (opiate)-binding or drug receptors were discovered in the mammalian brain, leading to global research on endogenous morphine-like substances. These were first discovered by John Hughes et al. from the Hans Kosterlitz laboratory at Aberdeen University in 1975 (3). In the same year, our team began researching endogenous morphine-like substances in bovine brains. In the beginning, we adopted a bioassay system that was similar to that used by the Kosterlitz's group to observe the inhibitory effects of the studied extracts on ileum contraction, an adverse action related to constipation due to morphine, caused by electrical stimulation. As unidentified compounds that inhibited ileum contraction always masked the effects of naloxone-reversible morphine-like substances, we discontinued the use of this strategy. Instead, we adopted the direct measurement of naloxone-reversible analgesic action (principal action of morphine) of fractionated substances following an intracisternal (*i.cist*.) injection, which was developed in-house (4). Through intensive studies, we successfully identified the naturally occurring neurodipeptide kyotorphin (tyrosine-arginine) that had opioid-like analgesic action (5). Further, we proved that kyotorphin acts as a neurotransmitter/neuromodulator in the mammalian brain. This review describes relevant historical studies and discusses the physiological roles and translational potential of kyotorphin, as reported in recent studies.

## Discovery of Kyotorphin

**Figure 1:**
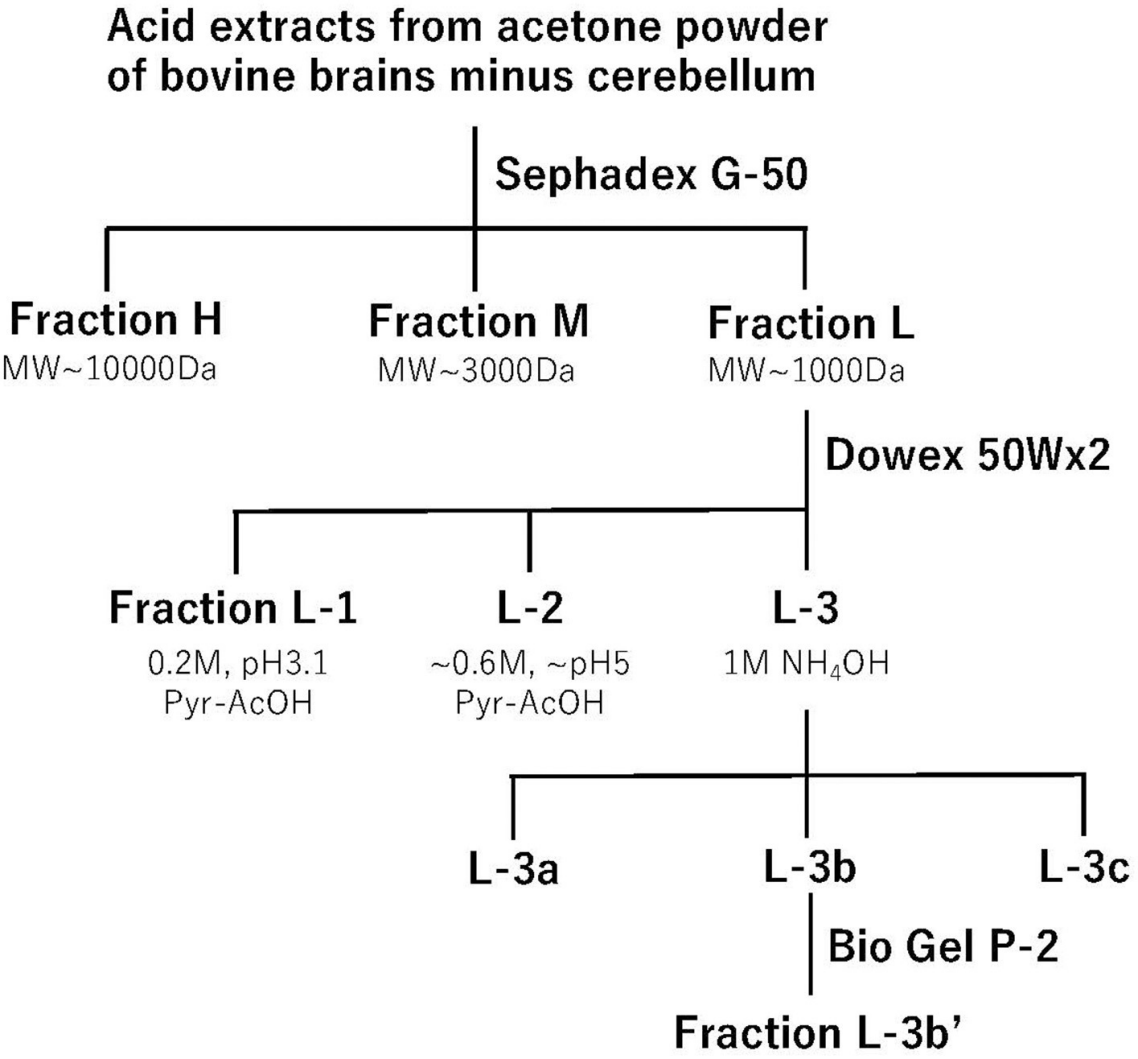
Step-wise purification of kyotorphin. Acid extracts from acetone powder of bovine brains minus cerebellum were applied to a Sephadex G-50 gel filtration chromatography. Opioid-like analgesic activity was observed in three fractions, and the materials in low molecular fraction (Fraction L) were then separated by a Dowex 50Wx2 cation exchange chromatography. Among 28 fractions, opioid analgesic activity was observed in 5 fractions. Among them, the basic fraction L-3b showed the most potent and relatively long-lasting opioid-like analgesic activity. The material L-3b' separated by the final BioGel P-2 gel-filtration chromatography was used for the amino acid sequence analysis.

As the major sites of morphine analgesia are located in the lower brain stem, including the periaqueductal gray matter (6) and nucleus reticularis (para)giganto cellularis, NRGC/NRPG (7, 8), we developed and chose the *i.cist*. administration method (4) for the assessment of analgesic effects. For the assessment of anti-nociceptive activity, we adopted the tail-pinch test using 500 g pressure adjusted artery clip in mice (9). In the test, as mice try to remove the clip, the nociceptive response does not reflect a simple spinal reflex; it is more indicative of central pain processing. Most importantly, we evaluated the reversal of the analgesic activity by naloxone, a morphine antagonist. Using these assays, we performed an analgesic evaluation of samples fractionated using several chromatographic techniques (5, 10, 11), as shown in Figure 1. Among these chromatographies, the most important separation of active substances was performed with Dowex 50Wx2 cation exchange chromatography, where 60–80% of analgesia was observed with the fractions L-1, L-2, L-3a, L-3b, and L-3c among 28 fractions separated using fluorescamine-labeled peaks, and complete naloxone antagonism was observed for fractions L-1, L-3b, and L-3c. The final fraction L-3b′ from Bio Gel P-2 shows as single compound in thin-layer chromatography and high-voltage paper electrophoresis (10). After amino acid analysis and dansylation to determine the N-terminal amino acid, the compound was revealed to be a dipeptide, tyrosine-arginine, with the same Rf value as the synthetic compound developed with high-performance liquid chromatography (HPLC). In this way, we were able to isolate opioid-like analgesic peptides using only classical chromatographic techniques. This success may be attributed to the nature of the aromatic and alkaline (basic) peptide that has a unique Dowex 50Wx2 profile. However, the *in vivo* bioassay to evaluate the naloxone-reversible central analgesic activity was a more laborious process than the *in vitro* bioassay using isolated organs (11).

## Molecular, Physiological, and Pharmacological Characterization of Kyotorphin

To address the key question of physiological roles of dipeptide kyotorphin, we firstly performed several experiments to determine its characterization as a neurotransmitter or neuromodulator.

### Regional and Subcellular Distribution in the Brain

Kyotorphin level in the rat brains was measured using HPLC with an electrochemical detector (12), which could detect aromatic residues (e.g., tyrosine). When the rat brain was divided into eight parts, according to the method proposed by Glowinski and Iversen (13), the kyotorphin concentration (ng/g tissue) was 719.5 in the midbrain, 556.5 in the pons and medulla oblongata, 391.8 in the hypothalamus, 367.1 in the cerebral cortex, 119.3 in the thalamus, 101.8 in the cerebellum, 61.8 in the hippocampus, 45.5 in the striatum, 405.1 in the dorsal half of the spinal cord, and 230.2 in the ventral half of the spinal cord. Thus, it seems that kyotorphin levels are relatively high in the regions related to the sites of action of morphine analgesia (PAG in the midbrain and NRGC/NRPG in the medulla oblongata) and the pain pathway (the dorsal horn in the spinal cord, PAG, hypothalamus, and cerebral cortex). It was also found that peripheral tissues such as the pituitary (11) and adrenal glands (14) have significant amounts of kyotorphin, suggesting that kyotorphin may play a role in peripheral organs. Furthermore, kyotorphin was also found in the brain of various mammals.

Regarding subcellular localization, kyotorphin was found to be concentrated in the synaptosomal fraction, which contains nerve-ending particles (15). The concentrations and contents of kyotorphin in various fractions (ng/mg protein, % of the total) were as follows: nuclear P1 fraction (1.27, 7.4); crude mitochondrial P2 fraction (5.87, 92.2); and microsome + cytosol S2 fraction (0.02, 0.4). Further, sucrose density gradient sub-fractionation of the P2 fraction showed the uneven distribution of kyotorphin concentration: 0.32–0.8 M in the myelin fraction (0 ng/mg protein); 0.8–1.2 M in the synaptosome fraction (17.1 ng/mg protein); and 1.2 M in the mitochondrial pellet fraction (0.78 ng/mg protein).

### Biosynthesis

#### Tyrosyl tRNA Synthetase Pathway

Unlike most neuropeptides that are produced by the processing of precursor peptides, kyotorphin is produced from tyrosine and arginine by the action of a specific kyotorphin synthetase (16, 17). While studying kyotorphin release from brain slices, we found that kyotorphin accumulates in a time-dependent manner in the presence of bestatin, an aminopeptidase inhibitor (18). Extensive accumulation of kyotorphin was also observed in the synaptosome fraction. We discovered kyotorphin synthetase, which produces kyotorphin from tyrosine and arginine in the presence of ATP and MgCl_2_. In a study on the purification of kyotorphin synthetase (17), we performed a radioimmunoassay using a specific kyotorphin antiserum. The starting materials, P2 crude synaptosomes, were lysed and the supernatant (synaptosol) was separated using Sephacryl S-300 gel-filtration, DE52 anion-exchange chromatography, and TSKgel G4000SW chromatography, in which active materials were observed at 240–250 kDa. From the enzymatic characterization, it was revealed that the optimal pH was 7.5–9.0, and enzymatic activities reached a plateau at 0.2–0.5 mM of tyrosine, 4–8 mM of arginine, 2–4 mM of ATP, and 2–4 mM of MgCl_2_. The Km values for tyrosine, arginine, ATP, and MgCl_2_ were 25.6, 926, 294, and 442 μM, respectively. The possible reaction mechanism of kyotorphin synthetase may be outlined as follows: tyrosine + arginine + ATP→ (synthetase, Mg^2+^)→ tyrosine-arginine (kyotorphin) + AMP + bisphosphate. The regional and subcellular distribution of kyotorphin synthetase in the brain (17) correlated well with that of kyotorphin content (12, 15). Enzyme activity was highest in the midbrain and medulla oblongata and in the synaptosome fractions. However, molecular characterization of kyotorphin synthetase has not been conclusively determined. A previous study demonstrated that *Bacillus stearothermophilus* tyrosyl-tRNA synthetase (TyrRS) could catalyze the *in vitro* synthesis of kyotorphin (17, 19), whereas *Streptomyces septatus* aminopeptidase could not synthesize kyotorphin from substrate amino acid; however, it resulted in several other dipeptides (20). Recently, we successfully demonstrated that tyrosyl-tRNA synthetase (TyrRS) is a potential kyotorphin synthetase in mammals (16). In this study, the Km of tyrosine and arginine for *in vitro* kyotorphin synthesis by recombinant human TyrRS (hTyrRS) was 200 and 1,400 μM, respectively, similar to that of partially purified kyotorphin synthetase from rat brains. Although the *in vitro* cell-free study showed that hTyrRS could produce several tyrosine-containing dipeptides (kyotorphin, tyrosine-tyrosine, tyrosine-proline, and tyrosine tryptophan), treatment of PC12 cells with siRNA antisense for TyrRS selectively blocked the *in vitro* biosynthesis of kyotorphin, but not that of tyrosine-tyrosine, tyrosine-proline, or tyrosine tryptophan. However, siRNA treatment did not affect cell survival or proliferation. In addition, TyrRS mRNA expression was higher in the midbrain and medulla oblongata than in other brain regions, which is consistent with the localization of kyotorphin synthetase activity in rat brains (17).

#### Calpain-Mediated Calpastatin Processing

Regarding kyotorphin biosynthesis, there is a possibility that kyotorphin is also produced from precursor polypeptides. When the soluble fraction from synaptosomes was incubated in the presence of bestatin, an amino peptidase inhibitor, kyotorphin accumulated in a time-dependent manner, possibly through calcium-activated neutral proteases (μ- and m-CANP),calpain-1 or calpain-2 (21). Detailed studies further demonstrated that the enzyme purified using DE52 cellulose, Ultrogel AcA, thiopropyl-Sepharose 6B, second DE52 cellulose, Ultrogel AcA34, and blue Sepharose CL-6B was characterized as a 74 kDa protein, a novel type of calpain lacking caseinolytic activity, with calpastatin identified as the substrate for this enzyme or precursor of kyotorphin (22). As the activation of this new type of calpain and calpain-1 requires more than 1 μM Ca^2+^, which is higher than the concentration at resting state (0.1–0.3 μM), this calpain-calpastatin system may be driven when neurons are activated. However, the exact physiological mechanisms are unclear.

### Kyotorphin Release From Synaptosomes

**Figure 2:**
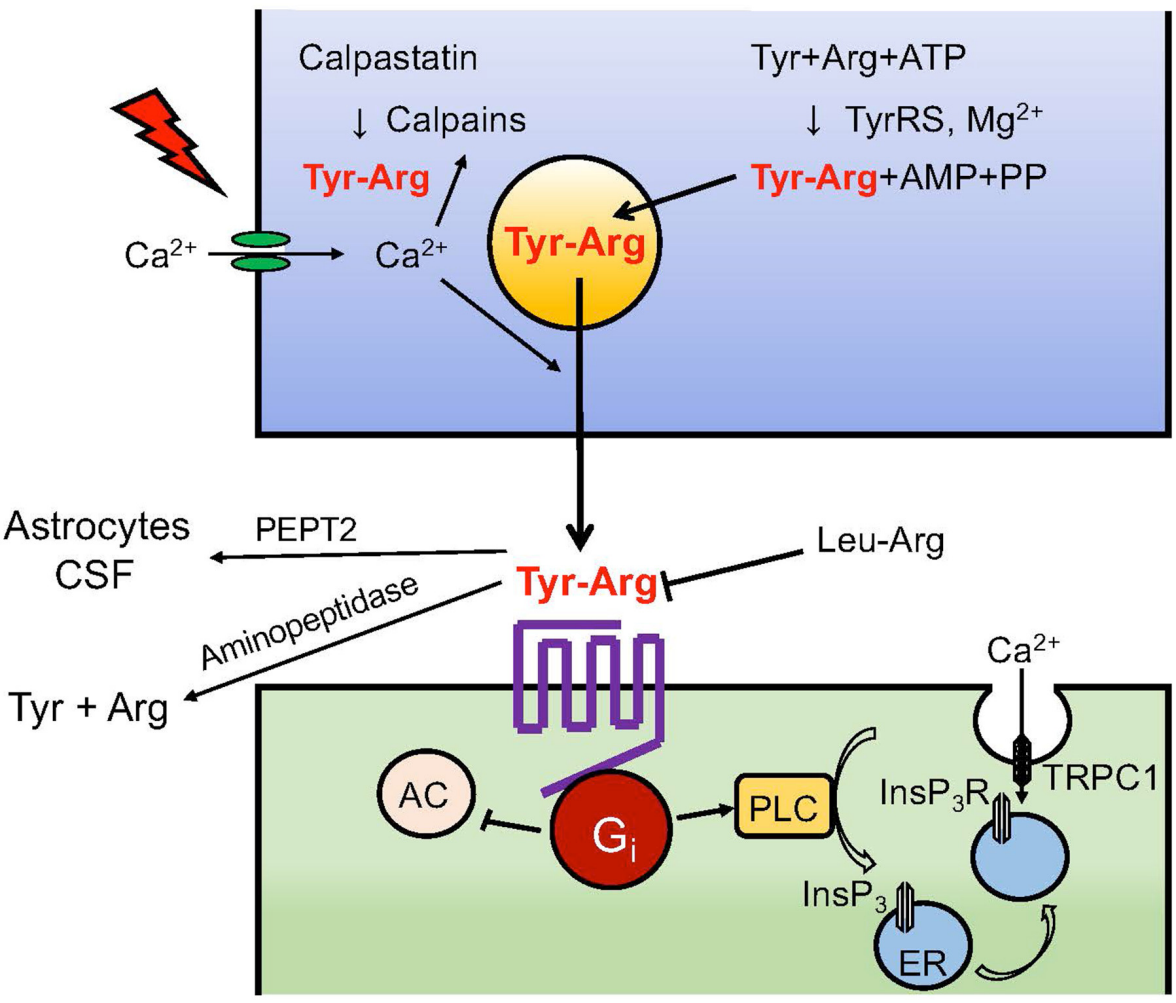
Neurotransmission of kyotorphin. Details of TyrRS-mediated kyotorphin (Tyr-Arg) biosynthesis, depolarization-induced Ca^2+^-dependent kyotorphin release, kyotorphin receptor-G_i_ coupling, PLC-InsP_3_R signaling, and inactivation system through degradation and excretion are described in the text. Kyotorphin receptor-mediated InsP_3_R activation may lead to a conformational coupling to TRPC1-dependent plasma membrane channels (24).

Although only limited information can be obtained regarding kyotorphin release from brain preparations, we have demonstrated unique findings that kyotorphin is incorporated into crude synaptosomes and released due to high K^+^ depolarization in a Ca^2+^-dependent manner (23). In this study, rat brain crude synaptosomes (P2 fraction) were incubated with 100 μM kyotorphin for 4 min at 37°C to allow active uptake in an energetic inhibitor-reversible manner. Kinetic analysis revealed that the Km and Vmax of kyotorphin uptake were 1.31 ± 0.12 × 10^−4^ M and 5.9 ± 0.5 pmol/mg protein/min, respectively. The fact that the supposed transporters show low affinity to kyotorphin and high capacity suggests that the mode of uptake is likely mediated through amino acid-type transporters and not by endocytosis through specific high-affinity kyotorphin receptor binding (Kd: 0.34 nM, Bmax: 36 fmol/mg protein), as described later. Details of the transporters involved in the kyotorphin uptake are discussed later, but this finding provides satisfactory evidence for the action of kyotorphin in neurons. When the synaptosome preparation, which had been filled with exogenous kyotorphin, was provided high K^+^ (50 mM) stimulation, approximately 35% kyotorphin was released in a Ca^2+^-dependent manner (23). These findings also indicate that kyotorphin is released from the neuronal endings (Figure 2).

### Receptor Signaling

#### Specific Kyotorphin Binding in Brain Membrane Preparations

To detect the specific binding of kyotorphin to brain membranes, we prepared ^3^H-kyotorphin possessing high specific activity using *B. stearothermophilus*-derived TyrRS, which was immobilized on Sepharose 4B (19, 25). The enzymatic reaction was performed by incubation of ^3^H-tyrosine with high specific activity and high concentration of arginine in the presence of 20 mM ATP and MgCl_2_ at 45°C for 48 h. After HPLC purification, ^3^H-kyotorphin was separated from other substrates. As ^3^H-kyotorphin non-specifically binds to glass-fiber filters, we adopted the centrifugation method to separate the membrane-bound form of ^3^H-kyotorphin from the free ^3^H-kyotorphin. From the Scatchard plot, it was revealed that brain membranes have high-affinity and low-affinity binding sites for ^3^H-kyotorphin. The Kd and Bmax were 0.34 nM and 36 fmol/mg protein for the high-affinity binding sites, whereas they were 9.07 nM and 1.93 pmol/mg protein for the low-affinity binding sites. The high-affinity binding using 0.5 nM ^3^H-kyotorphin in various brain regions was 121 (fmol/mg protein) in the hypothalamus, 104 in the amygdala, 77 in the occipital cortex, 67 in the frontal cortex, 65 in the thalamus, 58 in the medulla oblongata, 57 in the hippocampus, 53 in the midbrain, 48 in the striatum, and 39 in the cerebellum, indicating that there is some similarity in terms of regional distribution between ^3^H-kyotorphin binding and kyotorphin contents (12) or kyotorphin synthetase levels (17). In the competition study, the IC_50_ value was calculated for 20.8 (nM) for kyotorphin, 11.2 for leucine-arginine, 12.7 for phenylalanine-arginine, 37.6 for tyrosine-leucine, 224 for tyrosine-lysine, and >1,000 for other dipeptides, suggesting that ^3^H-kyotorphin binding recognizes dipeptides containing tyrosine or arginine.

#### Leucine-Arginine (Leu-Arg) Antagonizes Kyotorphin-Enhanced GTP-Binding Protein Activity

As ^3^H-kyotorphin binding was blocked in the presence of GTPγS + MgCl_2_, the receptor was presumed to couple with GTP binding proteins (25). When the low *Km* GTPase activity in the brain membrane preparation was measured, kyotorphin showed a concentration-dependent enhancement of this activity in the range of 10 nM to 100 μM (25). As L-leucine-L-arginine (Leu-Arg) did not activate the low *Km* GTPase activity, but it showed a strong inhibition of ^3^H-kyotorphin binding, Leu-Arg was presumed to be an antagonist for the GTP-binding protein coupled receptor (GPCR) for kyotorphin. This speculation was proven by the finding that the addition of Leu-Arg eliminated kyotorphin-induced enhancement of low *Km* GTPase activity (25).

#### G_i_-Coupling in Kyotorphin Receptor-Mediated Phospholipase C Activation

Since receptor-mediated enhancement of low *Km* GTPase activity is often reported in the case of G_i/o_- or G_s_-coupled receptors, but not for G_q/11_-coupled receptors, we speculated that G_i/o_ could be a possible candidate for coupling with kyotorphin receptor. Based on the analogy of μ-opioid receptor-G_i/o_ coupling (26, 27), we first treated the brain membranes with pertussis toxin, which blocks the interaction of receptors to G_i/o_ by ADP-ribosylation of cysteine SH-residue at the fourth amino acid from the C-terminal of the α-subunit (25). Pertussis toxin-ablated kyotorphin-enhancement of low *Km* GTPase activity was recovered by reconstitution with purified G_i_ or G_o_. However, as an effector system, we successfully observed that kyotorphin activated phospholipase C (PLC) in an experiment measuring inositol (1,4,5)-P_3_/InsP_3_ (25). The kyotorphin-induced and Leu-Arg-reversible PLC activation in the synaptosome membranes was also eliminated by pretreatment with pertussis toxin, and recovered by reconstitution with purified G_i_, but not G_o_. The pioneering finding that G_i_-coupled receptor mediates PLC activation (Figure 2) was supported by successive studies, and the concept has been further refined by a recent study (28), where the Gα_i_-Gβγ-PLCβ-Ca^2+^ signaling module was found to be entirely dependent on the presence of active Gα_q_. In other words, G_i_-mediated PLC activation was positively regulated by G_q_-coupled receptor activation.

#### Kyotorphin-Mediated Ca^2+^ Influx Through InsP_3_ Receptor

Based on the kyotorphin signaling through the activation of the G_i_-PLC system, we concluded that kyotorphin caused a G_i_-PLC-InsP_3_ receptor (InsP_3_R)-mediated transport of ^45^Ca^2+^, which had been filled in the resealed vesicles made of synaptosomal membranes (29). In this experiment, lysed synaptosomes were incubated with ^45^CaCl_2_ to generate inside-out and outside-out types of resealed vesicles containing ^45^CaCl_2_. When the resealed vesicles were placed on the GF/C filter and perfused with buffer, the basal ^45^Ca^2+^ release became stable after 10–20 min. The addition of InsP_3_ or kyotorphin (+GppNHp) caused an increase in ^45^Ca^2+^ release from inside-out or outside-out vesicles, respectively. The preceding treatment of synaptosomal membranes with pertussis toxin blocked the kyotorphin-induced ^45^Ca^2+^ release, but this effect was reversed by reconstitution of pertussis toxin-treated membranes with purified G_i_ reversed. Although the experiment demonstrated ^45^Ca^2+^ release from ^45^Ca^2+^-preloaded vesicles, kyotorphin signaling through the activation of G_i_-PLC-InsP_3_R under physiological conditions could cause Ca^2+^ influx from the extracellular space (mM Ca^2+^) into the cytosol (sub-μM Ca^2+^). This unique mechanism of GPCR-PLC-InsP_3_R-mediated Ca^2+^ influx was further explained by a working hypothesis that InsP_3_-mediated ^45^Ca^2+^ influx is driven by the conformational coupling of vesicular InsP_3_R and transient receptor potential C1 (TRPC1) in the plasma membrane (24), as seen in Figure 2.

### Kyotorphin Inactivation

To prove that an endogenous substance, kyotorphin is a neurotransmitter, as in the cases of glutamate, GABA, acetylcholine, dopamine, noradrenaline, and serotonin, the following criteria should be fulfilled: (1) storage and/or biosynthesis in the nerve terminals; (2) release upon neural stimulation; (3) specific receptor and possible antagonist; (4) post receptor signaling; and (5) rapid inactivation (signal turn-off system) through degradation or reuptake. As mentioned above, kyotorphin nearly fulfills all these criteria. In the case of established neuropeptides, enzymatic degradation is known to be the major inactivation mechanism. Similarly, the degradation of kyotorphin would also be the most probable inactivation mechanism. In a study, kyotorphin was rapidly degraded by incubation with diluted suspensions of rat brain homogenates (30). The apparent maximum rate Vmax and Michaelis' constant Km were 29.4 nmol/mg protein/min and 16.6 μM, respectively. Further, studies showed that kyotorphin degradation was effectively inhibited by the membrane-bound aminopeptidase inhibitor bestatin and the thiol protease inhibitor p-chloromercuribenzoate. Its degradation was also weakly inhibited by a lipophilic chelating agent (1,10-phenanthroline), but not inhibited by hydrophilic chelating agents (EDTA, nitriloacetic acid), serine protease inhibitors, or puromycin. The inhibitory constant K_i_ of bestatin was 0.1 μM, as derived from the Lineweaver-Burk plot. Co-administration of 50 μg bestatin (*i.cist*.) significantly potentiated the analgesic effects of kyotorphin (*i.cist*.), and the analgesia was eliminated by pretreatment with naloxone at 0.5 mg/kg, *s.c*. The degradation of kyotorphin by aminopeptidases has been also reported in other studies (31, 32). Akasaki et al. (33) purified kyotorphin degrading aminopeptidase (KTPase) to a homogeneity at 67 kDa protein, which mimics the similar enzyme inhibitor profile as previously reported bestatin-sensitive aminopeptidases in brain homogenates (30). Several kyotorphin derivatives, which are enzymatically stable, have more potent and longer analgesic actions than kyotorphin (34–37). An increasing number of reports also suggest an alternative inactivation mechanism wherein kyotorphin is excreted into the cerebrospinal fluid (CSF) or taken up by the astrocytes through a transporter PEPT2 (38). Thus, it is plausible that both enzymatic degradation and PEPT2-mediated excretion play roles in the *in vivo* inactivation (signaling turn-off) system for kyotorphin neurotransmission (Figure 2).

### Pharmacological Mechanisms Underlying Kyotorphin-Induced Opioid-Like Analgesic Effects

Although kyotorphin does not suppress opioid receptor binding and causes analgesic effects in a naloxone-reversible manner (5, 39), it was proposed that the opioid-like analgesic effects are mediated through endogenous opioids. Previous and more recent studies have suggested that kyotorphin releases Met-enkephalin from brain slices (5, 40), and that N-methyl tyrosine-arginine (NMYR), which is potent kyotorphin derivative (37) induces a potent analgesia in a manner of reversal by the genetic deficiency of endogenous opioid peptide precursors, preproenkephalin or proopiomelanocortin (41). Although the possibility cannot be excluded that kyotorphin inhibits the enzymatic degradation of Met-enkephalin, its contribution to the opioid-like analgesic effects seems to be negligible (32, 42).

#### Kyotorphin-Induced Met-Enkephalin Release

To measure Met-enkephalin release, guinea pig striatal slices of 500 μm thickness or 200 μm cubic slices of the whole spinal cord were placed in a 1.5 ml perfusion chamber (40). The perfusion from the bottom to the top was conducted at 37°C at a rate of 1 ml/min with Krebs-bicarbonate medium gassed with 95% O_2_ and 5% CO_2_, and perfusion samples were collected at 3-min intervals. The addition of 50 mM KCl caused an ~60-fold Met-enkephalin release, compared to the basal release, but it was completely eliminated by the omission of CaCl_2_. The addition of 1 or 10 μM kyotorphin caused a 1.6- or 3.4-fold increase in Met-enkephalin release, respectively, and kyotorphin-induced release was also stopped by the removal of CaCl_2_. The kyotorphin-induced Met-enkephalin release was also stopped by the addition of 2 μM tetrodotoxin, which itself slightly reduced the basal release, suggesting that striatal preparation may be spontaneously stimulated by excitatory transmitters such as glutamate leaked from damaged neurons during the slice preparation. When the striatal slices were electrically stimulated at 10 Hz by two disc electrodes fixed to both the top and bottom, a two-fold Met-enkephalin release was observed, and the further addition of 1 μM kyotorphin showed a 3.6-fold increase. However, it remains unclear whether kyotorphin potentiates the electrical stimulation-induced release of Met-enkephalin, since kyotorphin only shows additive effects (1.6 + 2.0 = 3.6-fold) to the electrical stimulation-induced Met-enkephalin release. In the experiments using spinal cord preparations, 10 μM kyotorphin caused a 2.2-fold increase in Met-enkephalin release (40), suggesting that the action of kyotorphin is also observed in the preparation of the spinal cord, while the potency of Met-enkephalin release is slightly weaker.

#### L-Tyr-D-Arg-Induced Met-Enkephalin Release

The addition of 10 μM L-Tyr-D-Arg (Tyr-D-Arg), an enzymatically stable kyotorphin derivative, caused an increase in Met-enkephalin release to the same level as kyotorphin (35). As Tyr-D-Arg showed more potent analgesia in the tail pinch test (ED50: 6.2 nmol/mouse, 60-min duration by 29.5 nmol) than kyotorphin (ED50: 15.7 nmol/mouse, 30-min duration by 59.2 nml) (36), the enhanced analgesic potency of Tyr-D-Arg seems to be attributed to the enzymatic stability, but not to the potency of Met-enkephalin release. Similar potency of Met-enkephalin release by kyotorphin and Tyr-D-Arg was reported by other investigators using the preparation of rat hypothalamus, in which both peptides showed no release of preloaded [^3^H]noradrenaline, [^3^H]GABA, or [^3^H]D-aspartate (43).

#### Possible Molecular Mechanisms Underlying Kyotorphin-Induced Met-Enkephalin Release

The mechanisms underlying kyotorphin-induced Met-enkephalin release or opioid analgesia remain unknown. To date, several studies have demonstrated kyotorphin-induced Met-enkephalin (or endogenous opioids) release from brain or spinal cord preparations (35, 40, 43). In addition to the direct evidence, electrophoretically charged kyotorphin caused an excitatory effect on the spontaneous activity of a neuron in the medulla NRPG, a site of morphine analgesia in a naloxone-reversible manner (44). However, in the peripheral system, kyotorphin and Tyr-D-Arg increased the amplitude and mean quantal content of the fast excitatory postsynaptic potentials in the isolated sympathetic ganglia with preganglionic nerves, and these responses were reversed by naloxone (45). It was also interesting to note that kyotorphin inhibited the isoprenaline-induced increase in the twitch tension of the ventricular papillary muscle of rats, and this effect was eliminated by Leu-Arg and naloxone (46).

**Figure 3:**
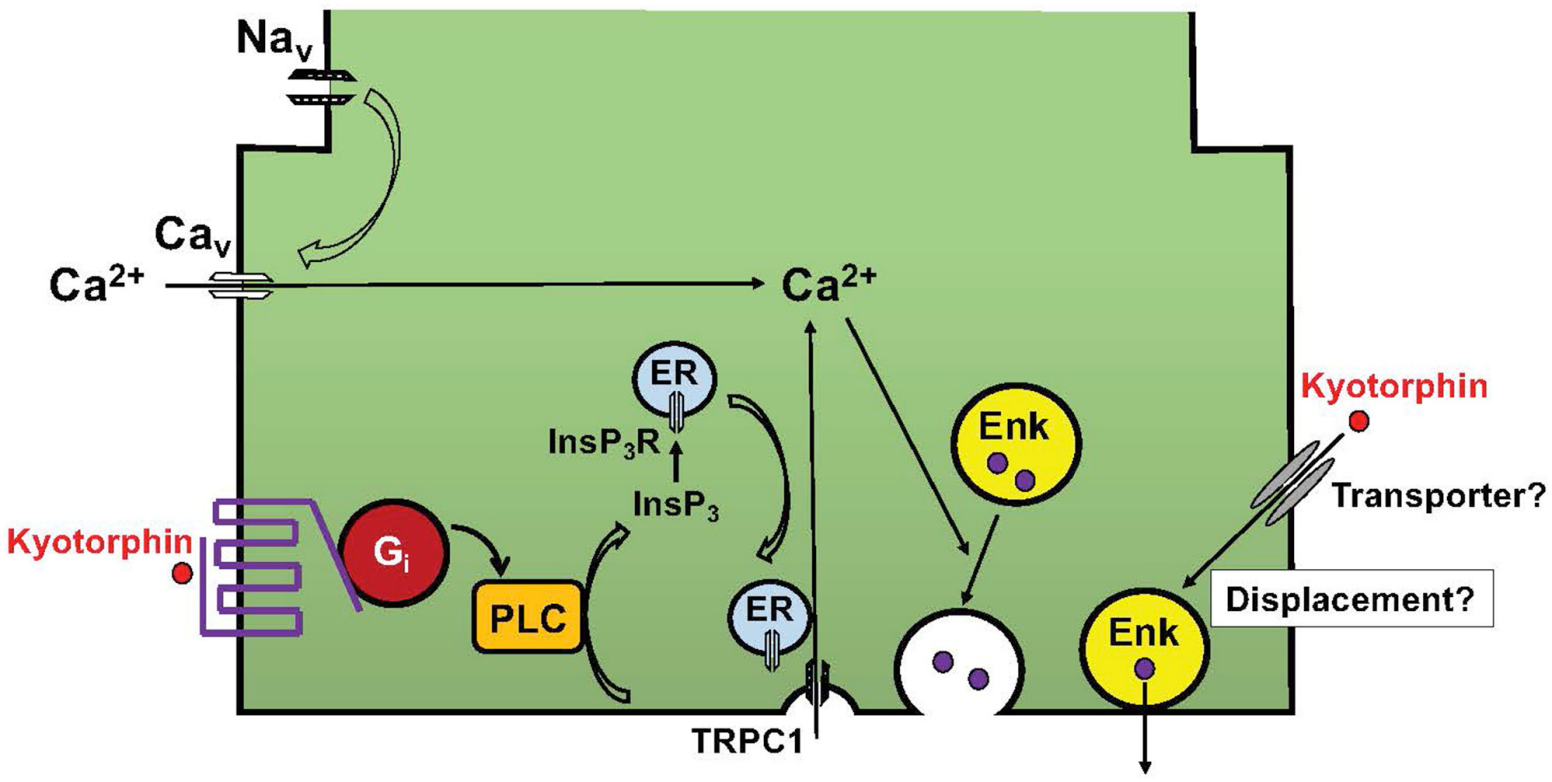
Working hypotheses for the mechanisms underlying kyotorphin-induced Met-enkephalin release. The kyotorphin (Tyr-Arg) receptor mediates the activation of G_i_ and PLC. This signaling leads to the production of InsP_3_ and activation of InsP_3_R in the endoplasmic reticulum (ER). The conformational coupling of InsP_3_R with TRPC1 opens plasma membrane Ca^2+^ channels (24). This receptor-operated mechanism may enhance the recruitment of Met-enkephalin-containing synaptic vesicles to the plasma membranes. Although no evidence is available, but it is interesting to speculate that kyotorphin is incorporated into nerve terminals, and thereby displaces and excretes Met-enkephalin from synaptic vesicles attached to the plasma membranes.

In the receptor system, it was found that kyotorphin couples with G_i/o_-coupled receptors (25), as seen in the case of the μ-opioid receptor (26). Although G_i/o_ is generally related to inhibitory functions through G_i_-mediated adenylyl cyclase inhibition and G_o_-mediated inhibition of voltage-dependent calcium channels (47), the kyotorphin receptor mediates the activation of G_i_, PLC, and Ca^2+^ signaling in G_i_ reconstitution experiments (25). A recent report supported the G_i_-mediated activation of PLCβ-Ca^2+^ signaling and also demonstrated that this module is dependent on the presence of active Gα_q_ (28). Furthermore, it has been reported that GPCR-PLC-InsP_3_ activation mediates Ca^2+^ influx through the conformational coupling of vesicular InsP_3_R and plasma membrane TRPC1 (24, 29), as seen in Figures 2, 3. Although the omission of Ca^2+^ in the perfusion medium abolished the kyotorphin-induced release of Met-enkephalin from brain slices (40), it remains unclear whether kyotorphin receptor-induced Ca^2+^ influx through a G_i_-PLC-InsP_3_R mechanism contributes to the Met-enkephalin release. As the kyotorphin-induced Met-enkephalin release was abolished by the presence of tetrodotoxin or the omission of Ca^2+^ in the perfusion medium (40), the activation of tetrodotoxin-sensitive voltage-dependent Na^+^ channels is presumably mediated by glutamate or other excitatory substances leaked from the damaged brain preparations, but not by kyotorphin through G_i_-coupled receptors. However, it would be interesting to examine whether kyotorphin-induced Met-enkephalin release requires both the G_i_-PLC-InsP_3_R mechanism and physiologically occurring depolarization of nerve terminals.

Finally, it is not understood how kyotorphin induces the release of Met-enkephalin, but not other transmitters such as preloaded [^3^H]noradrenaline, [^3^H]GABA, or [^3^H]D-aspartate (43). Although no evidence is available, it has been speculated that kyotorphin is incorporated through unidentified transporters and subsequently displaces Met-enkephalin in synaptic vesicles, causing its release (Figure 3), as seen in the case of tyramine-induced noradrenaline release (48). Although kyotorphin is reported to be an endogenous substrate of the peptide transporter PEPT2, this transporter mechanism is unlikely to be involved in kyotorphin-induced Met-enkephalin release from neurons, since PEPT2 is involved in the clearance of enkephalin from the brain (49).

## Translational Research

### Arginine-Induced Opioid Analgesia Due to Kyotorphin Production

As stated above, kyotorphin meets most of the criteria for a neurotransmitter/neuromodulating peptide and shows opioid-like analgesia in rodents. In addition to these findings, it has been reported that kyotorphin is detected in the CSF of patients with persistent pain (50), suggesting that *in vivo* levels of kyotorphin may be partially related to the pain threshold in humans. However, the fact that the Km of substrate arginine for kyotorphin synthetase or TyrRS at 926 or 1,400 μM (16, 17) is much higher than that in the human plasma at 50–100 μM (51) indicates the possibility that arginine administration elevates brain kyotorphin levels. Indeed, the oral administration of arginine at 1 g/kg increased kyotorphin levels in the midbrain and medulla oblongata (16), where sites of morphine analgesia are located. In the thermal nociception test, arginine at 0.1, 0.3, and 1 g/kg (p.o.) showed dose-dependent analgesia, and analgesia with 1 g/kg (p.o.) was abolished by 3 fmol of N-methyl Leu-Arg (NMLR), a kyotorphin receptor antagonist (37). As the arginine-induced analgesia was also abolished by 0.1 nmol of naloxone (*i.c.v*.), and significantly attenuated in preproenkephalin- or proopiomelanocortin-deficient mice (41), it is suggested that systemic administration of arginine produces kyotorphin in the brain, leading to causing opioid-like analgesia *via* a release of endogenous opioids. The evidence for arginine-induced analgesia has also been reported by other investigators (52). As arginine has been found to mediate gastrointestinal relaxation (1) as well as kyotorphin production and subsequent opioid analgesia, the combined treatment of arginine may reduce the dose of morphine for pain relief in terminal cancer patients, which would in turn reduce morphine-induced constipation.

### Development of Kyotorphin Derivatives

Kyotorphin derivatives that are enzymatically stable, permeable across the blood-brain-barrier, and function added (conjugated) have been reported in literature (34, 36, 37, 53–55). The initial kyotorphin derivative to be less susceptible to enzymatic degradation was Tyr-D-Arg, which is not the substrate for purified KTPase (33) and showed 5.6 times more potent and longer-lasting (60 min) analgesic effects than the case with kyotorphin (30 min) when given *i.cist* (36). As the coadministration of bestatin potentiated the analgesia by kyotorphin, but not by Tyr-D-Arg (31), the derivatization of kyotorphin to avoid the degradation by KTPase would be beneficial for the analgesia. Following the study with Tyr-D-Arg, NMYR and Tyr-Arg-NH_2_ (KTP-NH_2_) were found to be potent in producing the analgesic activity in terms of systemic administration (34, 37, 41). The potent analgesic activities of these peptides are presumed to be attributed to the resistance to enzymatic degradation, though the biochemical evidence for the enzymatic resistance remains to be done. Additional mechanism would be their blood brain barrier (BBB) permeability. Castanho and his colleagues have demonstrated that there is a positive correlation between analgesic efficacy and relative permeability using the *in vitro* BBB model comprised of transwell apparatus and lipid membranes (34, 55). Although it remains elusive whether all these derivatives are mediated through a kyotorphin receptor, there is a report that the analgesic activity of NMYR administered through a systemic route (*s.c*.) was abolished by the *i.c.v*. injection of kyotorphin receptor antagonist, NMLR (41). This finding also indicates that NMYR is penetrated through BBB and exerts analgesia through the kyotorphin receptor in the brain. As Niwa and his colleagues developed a good transwell BBB model comprised of endothelial cells, pericytes and astrocytes (56), the more precise study of brain permeability using this *in vitro* BBB model as well as the direct *in vivo* pharmacokinetics and pharmacodynamics analysis of brain peptide levels using Liquid Chromatograph—Mass Spectrometry would be necessary for the development of better brain permeable compounds.

Furthermore, with function-added (conjugated) derivatives such as GABA-kyotorphin (57), kyotorphin-nitroxide (58) and ibuprofen-kyotorphin amide (IbKTP-NH_2_) (53, 54), the extent to which kyotorphin receptor mechanisms are involved in their analgesic action remains unclear. Among them, Tyr-D-Arg and NMYR would be authentic kyotorphin derivatives since Tyr-D-Arg showed Met-enkephalin release (35, 36) and NMYR-induced analgesia was blocked by the enzymatically stable NMLR (37, 41), which is a kyotorphin receptor antagonist. In addition, NMYR-analgesia was abolished by naloxone and significantly inhibited in preproenkephalin- or proopiomelanocortin-deficient mice (41). Interestingly, it has been reported that oral or intrathecal (*i.t*.) administration of the non-peptide anti-inflammatory drug 3-(difluoromethyl)-1-(4-methoxyphenyl)-5-[4-(methylsulfinyl)phenyl]pyrozole (FR14023) showed potent analgesic action that was blocked by Leu-Arg (*i.t*.). However, it is unclear whether FR14023 action is mediated by binding to the kyotorphin receptor or kyotorphin release/production *in vivo*.

### Other Pharmacological Applications

The translational research and pharmacological characterization of other applications in addition to opioid-like analgesic action for kyotorphin and its derivatives have been well-reviewed by Perazzo et al. (59). Among them, studies on the etiological roles of kyotorphin for Alzheimer's disease (AD) and its therapeutic potential can be considered important. Santos et al. (60) demonstrated that kyotorphin levels in the CSF of AD patients are lower than those in normal subjects, and there is an inverse correlation between kyotorphin and p-Tau, a marker of neurodegeneration found in the brains of AD patients. This study leads to translational studies showing that kyotorphin (*i.c.v*.) suppresses the streptozotocin (*i.c.v*.)-induced model of sporadic AD, which is characterized by increased locomotor activity, decreased level of anxiety, and impaired spatial and working memory (61). Another report demonstrated that the systemic administration of amidated kyotorphin (32.3 mg/kg, *i.p*.) reversed the memory impairment in an *i.c.v*. amyloid-β peptide (Aβ)-induced sporadic AD model in rats and Aβ-induced decrease in the spine density in cortical neuronal cultures (62).

There are also interesting reports demonstrating the beneficial anti-convulsant action of kyotorphin. Godlevsky et al. (63) reported that the administration of 2.5–10 nmol of kyotorphin in the left lateral ventricle, the bilateral CA1 hippocampi, or the reticular part of the substantia nigra of rats significantly inhibited picrotoxin (2 mg/kg, *i.p*.)-induced convulsive behaviors. Similarly, Bocheva and Dzambazova-Maximova (64) reported that 20 μg kyotorphin (*i.c.v*.) significantly suppressed pentylenetetrazole (85 mg/kg, *s.c*.)-induced seizures and antinociceptive effects in the tail flick and hot plate tests. This study also reported that the kyotorphin derivative, tyrosine-L-canavanine, which is a structural analog of L-arginine, had comparatively stronger effects.

Further, studies have suggested that kyotorphin derivatives have several beneficial properties such as anti-inflammatory and anti-microbial effects. KTP-NH_2_ decreased the number of rolling leukocytes in a mouse model of inflammation induced by lipopolysaccharide (42). Regarding antimicrobial effects, KTP-NH_2_ and IbKTP-NH_2_ induced membrane blebbing, disruption and lysis of *Staphylococcus aureus* in the experiment using atomic force microscopy (65), though it remains elusive whether these actions are mediated by the kyotorphin receptor. Furthermore, some studies have demonstrated that kyotorphin has harmful effects such as opioid receptor-mediated inhibition of heart rate (66) or twitch tension of cardiac muscles (46), stress response due to augmented release of oxytocin, and activation of the sympathetic nervous system, which increases blood pressure (67). If these harmful effects occur by the action of endogenous kyotorphin, kyotorphin receptor antagonists, such as NMLR may have beneficial actions.

## Conclusion and Future Perspective

A Pub-med search using kyotorphin as a keyword showed that 242 publications have been published in January, 2021. As a researcher involved in the discovery of kyotorphin, I would like to express my gratitude to all researchers for their great contributions in this area. Initially, it was thought that kyotorphin was only a residual product of protein degradation. However, the following findings have assuaged skepticism regarding the importance of this dipeptide: (1) kyotorphin is unevenly distributed in the brain, it is found in high concentrations in the pain pathway or the regions involved in morphine analgesia (12); (2) kyotorphin is subcellularly localized in the synaptosome fraction or nerve-ending particles (15); (3) a specific synthetase, TyrRS, generates kyotorphin from tyrosine and arginine using ATP (16, 17); (4) kyotorphin may be also processed from calpastatin by a novel calcium-activated neutral protease or calpain (22); (5) kyotorphin preloaded into the synaptosome is released by high K^+^ depolarization in a Ca^2+^-dependent manner (23); (6) kyotorphin has a specific receptor, which mediates the activation of PLC and inhibition of adenylyl cyclase through G_i_ (25); (7) Leu-Arg works as a specific kyotorphin receptor antagonist (25); (8) membrane-bound aminopeptidase or PEPT2-mediated excretion play a role in kyotorphin inactivation (30). The aforementioned findings suggest that kyotorphin meets the criteria for a neurotransmitter/neuromodulator. The additional important issues that need to be addressed are the cloning of the kyotorphin receptor and the molecular mechanism of enkephalin release, which has remained unclear since the discovery of kyotorphin. It is expected that future studies may be focused on exciting topics such as translational research in the field. As kyotorphin is a minimal biological peptide, development of function-added kyotorphin derivatives can be anticipated. These would include not only the brain-permeable kyotorphin derivatives replaced with modified amino acids, but also hybrid kyotorphin derivatives conjugated with small compounds, which have additional pharmacological actions. As studies have shown that kyotorphin is involved in the etiology or treatment of AD, epilepsy, inflammation, and chronic pain, further studies of the beneficial effect of kyotorphin derivatives are expected.

## Author Contributions

The author confirms being the sole contributor of this work and has approved it for publication.

## Conflict of Interest

The author declares that the research was conducted in the absence of any commercial or financial relationships that could be construed as a potential conflict of interest.
